# Exosome-Mediated Crosstalk Between Cancer Cells and Tumor Microenvironment

**DOI:** 10.3390/cells14221750

**Published:** 2025-11-08

**Authors:** Sara H. Saad, Alex Kashanchi, Mohammad Asad Zadeh, Anastasia Williams, Elena V. Batrakova

**Affiliations:** Laboratory of Molecular Virology, School of Systems Biology, George Mason University, Discovery Hall Room 182, 10900 University Blvd, Manassas, VA 20110, USA; ssaad5@gmu.edu (S.H.S.); akashanc4@gmail.com (A.K.); masadzad@gmu.edu (M.A.Z.); awill57@gmu.edu (A.W.)

**Keywords:** exosomes, extracellular vesicles (EVs), tumor microenvironment (TME), cancer progression, immune modulation, B cells, dendritic cells, tumor-associated macrophages (TAMs)

## Abstract

Exosomes are extracellular vesicles that play a central role in mediating intercellular communication within the tumor microenvironment (TME). Cancer-derived exosomes carry proteins, nucleic acids, and lipids that reshape the phenotype and function of surrounding stromal and immune cells, thereby promoting tumor progression, angiogenesis, metastasis, and resistance to therapy. At the same time, exosomes derived from TME components, including macrophages, dendritic cells, B cells, T cells, fibroblasts, neutrophils, and NK cells, reciprocally influence tumor growth and immune evasion. These bidirectional interactions highlight exosomes as both drivers of tumor progression and regulators of antitumor immunity. In this review, we synthesize current evidence on the diverse mechanisms by which exosomes reprogram immune and stromal cells, with a focus on their dual roles in cancer biology. We also discuss emerging therapeutic strategies to inhibit exosome biogenesis, release, and function, underscoring their translational potential as novel targets for cancer diagnosis and treatment.

## 1. Introduction

Cancer remains one of the leading public health concerns worldwide. It is estimated that in 2025, in the United States alone, there will be 2,041,910 new cancer diagnoses and 618,120 cancer-related deaths [1]. Cancer is a complex systemic disease that is associated with a combination of abnormal physiological processes (i.e., sustained angiogenesis, immune evasion, and chronic inflammation) [2]. Each of them has different characteristics, risk factors, and causes. This poses a challenge to understanding the underlying causes and, as a result, hinders the development of therapeutic strategies for cancer treatment. However, there is an increasing body of evidence that suggests in all cases that exosomes play a pivotal role in cancer biology, affecting fundamental cancer characteristics. This is a novel concept, as the understanding of interactions between tumor cells and their microenvironment was only established through cell-to-cell contacts. In this context, elucidation of the regulatory mechanisms of exosomes in tumors can uncover their roles in cancer progression and invasion, providing new ideas for the clinical treatment of cancers.

Extracellular vesicles (EVs) are membrane-bound particles released by nearly all cell types and play essential roles in intercellular communication. EVs are broadly classified into apoptotic bodies, microvesicles, and exosomes, each differing in size, origin, and cargo composition. Exosomes are a class of small, single membrane EVs that are known as critical messengers for intercellular communication between adjacent cells, as well as between cells and distant organs [3]. They carry various biologically active molecules including nucleic acids, proteins, lipids, and metabolites that reflect characteristics of their parental cells. Numerous studies revealed that exosomes are involved in almost all significant biological processes under normal physiological conditions, such as embryonic development, central nervous system (CNS) signaling, immunomodulation, tissue repair and regeneration, blood coagulation, and material metabolism [4,5,6,7]. Recently, it was discovered that exosomes are also involved in various cancer biological processes, such as pathogenesis, tumor development, progression, and metastasis of cancer [8]. Thus, exosomes serve as key couriers whose content and function are determined by their parental cells (e.g., healthy or cancerous), leading to varied and sometimes opposing biological outcomes. These properties make exosomes uniquely suited to influence the tumor microenvironment, affecting cancer growth, metastasis, and responses to therapy. Given their role in cancer biology, the remainder of this review focuses specifically on exosomes and their contributions to tumor development and therapeutic potential.

Tumor-derived exosomes carry oncogenic content that is different from the content of exosomes released by healthy normal cells, and the oncogenic content is further varied between different cancer cells. Furthermore, it has been shown that oncogenic content correlates with tumor development and responses to anticancer therapy [8]. In general, tumor-derived exosomes transport factors that promote growth, invasion, and development of drug resistance in cancer cells. These tumor-derived exosomes thus play an important role in the intercommunications between cancer cells and the tumor microenvironment (TME; composed of stromal tissue, immune cells, and extracellular matrix). For instance, tumor-derived EVs have been shown to carry immunosuppressive factors aimed at reprogramming the immune system. Interestingly, the number of tumor-derived exosomes in the plasma of cancer patients and the relative amounts of exosomes from cancer cells and normal cells vary. Nevertheless, tumor-derived exosomes represent most of all circulating exosomes in the case of advanced malignancies. This underscores the significant role of tumor-derived exosomes in cancer progression [9] and highlights how these exosomes and their cargos serve as potential therapeutic targets, prognostic markers, and carriers for anticancer drugs.

Exosomes released by non-cancerous cells within the TME actively interact with cancer cells, influencing tumor behavior through the transfer of signaling molecules and regulatory biomolecules. This bidirectional crosstalk regulates key processes including tumor growth, metastasis, immune evasion, and therapy resistance. Understanding how TME-derived exosomes communicate with tumor cells offer critical insight into the cellular networks that drive cancer progression and highlight potential avenues for therapeutic intervention.

In this review, we discuss the interplay between cancer cells and the TME, specifically in regard to the role of exosomes in cancer progression. Here, the involvement of different immune response cells and various types of tumor cells in these communications is described. We also underscore the importance of targeting this exosome-mediated crosstalk for anticancer therapy. In addition, we would like to point out several excellent reviews that describe the role of exosomes in cancer development [10,11,12].

## 2. Immune Modulation of Tumor Microenvironment (TME) by Cancer Cell-Derived Exosomes

Various exosome-mediated mechanisms that aim at inactivation of immune response and supporting cancer cell growth were reported [13,14,15]. Cancer cell-derived exosomes transfer specific ligands that modulate gene expression and, as a result, the functions of the immunocytes (summarized in Table 1). According to these investigations, cancer cell-derived exosomes are heterogeneous multi-signal messengers. They can affect different types of immunocytes, including T cells, monocytes, macrophages, myeloid-derived suppressor cells (MDSCs), dendritic cells, fibroblasts, B cells, and natural killer (NK) cells promoting tumor progression. In general, two main effects of cancer-released exosomes on the TME were found: (i) suppression of immune surveillance, and (ii) activation of procancer growth factors. Cancer-induced immune suppression includes apoptosis of T cells, differentiation and maturation of dendritic cells (DCs), and inactivation of NK cells. Activation effects include proliferation of T and B cells (Tregs and Bregs), differentiation of macrophages to M2 subtype, and proliferation of MDSCs.

### 2.1. Suppression of T Cells

The innate immune system is the body’s first line of defense against cancer, and T cells are crucial key players in the control of cancer. Under physiological conditions, T cells recognize tumor-associated antigens that are introduced by antigen-presenting cells (APCs) and destroy them. It was found that even in a healthy body some portions of new cells have the potential to become cancerous, but they are constantly eliminated by immunocytes, that are actively scanning for abnormal cells. However, when this system fails and cancer cells start proliferating, they release exosomes with different biologically active compounds that facilitate the reprogramming of cells of the immune system and the promoting tumor growth by angiogenesis. These exosomes can inhibit T cell activation through multiple mechanisms, such as expressing inhibitory molecules, interfering with signaling pathways, and metabolic reprogramming [50].

A considerable number of research studies indicate that cancer cells release exosomes loaded with different proteins that inhibit T cell activity [16,17,18,20,21,23,27,29,30,51,52]. The variety of these inhibitory proteins is staggering. Thus, exosomes released by head and neck cancer (HNC) cells carry elevated levels of several proteins on their surface, including TGF-β, FasL, OX40, OX40L, TRAIL, PS, NKG2DL, TβRII, and HSP70 that induce apoptosis and suppress T cell activation and proliferation [51]. Inhibitory effects of FasL-positive exosomes were also found in the case of prostate cancer and in oral squamous cell carcinoma [21,22]. Mechanistic studies revealed that FasL-containing exosomes interact with T cells, and induce caspase-3 cleavage and cytochrome C release. As a result, T cells display the loss of mitochondrial membrane potential, a key event in the apoptosis cascade, and a reduction in T cell receptor (TCR) zeta chain expression, which causes downregulation in T cell activation and responsiveness [53]. Consequently, the levels of FasL-positive exosomes in patients with oral squamous cell carcinoma correlated with tumor growth and nodal involvement [22]. Furthermore, exosomes released by highly metastatic breast cancer cells (4T1) directly suppressed T cell proliferation [23]. This resulted in the inhibition of the anticancer immune response in premetastatic organs and development of lung metastases. They can condition a favorable microenvironment that promotes metastatic colonization in the lungs.

Another well-documented immune-suppressive protein carried by cancer-derived exosomes is Programmed cell Death 1 ligand 1 (PD-L1) which travels to reach distant immune cells. The PD-L1 levels on the surface of exosomes are associated with tumor-facilitating growth and progression, and the prevention of the immune system’s ability to fight the tumor [16,17,20]. Thus, melanoma cell-derived exosomes were found to carry PD-L1 that inhibit tumor-specific cytotoxic CD8+ T cells and facilitate the evading of the immune system [20]. The mechanism of immunosuppressive effects of PD-L1-expressing exosomes includes the binding of PD-L1 to PD-1 expressed on T cells, reducing cytokine production and inducing apoptosis in CD8+ T cells [19,52]. Furthermore, it was reported that IFN-γ treatment upregulates PD-L1 expression on cancer exosomes and inactivation leads to tumor cell response to this treatment with an increase in PD-L1 on the exosomes [18]. This was recognized as an adaptive response to CD8+ T cell activation and their suppression by cancer-derived exosomes. In another study, tumor-derived exosomes with PD-L1 expressed on their surface were found in the draining lymph node [18]. The importance of this finding relates to the ability of PD-L1-containing cancer-derived exosomes to reach lymph nodes and systemically act to suppress T cell activation in the whole body, promoting tumor growth and metastasis at distant sites.

Mitzaei et al. found that stem-like brain tumor-initiating cells (BTICs) produce another immunosuppressive protein, tenascin-C, that inhibits T cell proliferation [27]. Importantly, cancer-derived exosomes that express high levels of tenascin-C on their surface transfer this protein to T lymphocytes and reduce mTOR signaling through interactions with α5β1 and αvβ6 integrins on T cells. The inhibitory role of tenascin-C released by tumor cells was found in many other cancer types, including prostate cancer [19,28,54], lung cancer [55], breast cancer [56], bladder cancer [57,58], pancreatic cancer [59,60], and colorectal cancer [61].

Next, CD8+ T cell exhaustion by cancer cell-released exosomes carrying thyroid-stimulating hormone receptor (TSHR) was reported in the case of colorectal cancer (CRC) [30]. T cell inhibition and CRC immune evasion were attributed to TSHR interaction with thyroid-stimulating hormone (TSH). Thus, TSH/TSHR signaling resulted in T cell exhaustion and tumors desensitizing to PD-1 and anticancer therapy. Furthermore, cancer-released exosomes carrying immunoregulatory protein galectin-1 (Gal-1) were shown to exert a potent suppressor function in CD8+ T cells [24]. Once more, this feature was found in several head and neck cancer-derived cell lines.

The inhibition of CD4+ T lymphocytes was also reported by cancer-derived exosomes. These exosomes downregulate differentiation and proliferation of CD4+ T cells that are necessary for activating CD8+ T cells to proceed in tumor destruction [29,62]. In addition, exosome-bound oncogenic mutant p53 inhibits glucose metabolism that promotes apoptosis in CD4+ T lymphocytes under metabolic stress. The decline in CD4+ T cell population resulted in the reduction in cytokines released into the TME, compromising antitumor immunity. Next, a special role in tumor progression of chaperonin-containing TCP1 subunit 2 (CCT2), a constituent of the CCT chaperonin complex, was reported in [63]. It was demonstrated that breast cancer cells release exosomes with incorporated CCT2 protein and suppress the activation and proinflammatory cytokine secretion of CD4+ T cells. According to functional analysis, CCT2-loaded exosomes facilitated cancer growth, as well as the dissemination of metastasis through the activation of the JAK2/STAT3 signaling pathway. Consequently, the decreases in CCT2 resulted in activation of CD4+ T cells and inhibition of cancer growth.

Along with oncogenic proteins, cancer cell-derived exosomes may carry various nucleic acids, such as mRNAs, microRNAs, and DNA fragments that cause gene regulation and inactivation of the immune system. Gastric cancer (GC)-derived exosomes were reported to carry various circular RNAs (circRNAs). One such example is vcircMAN1A2 (hsa_circ_0000118) that promotes GC proliferation and metastasis by suppressing the antitumor immunity of T cells [64]. Specifically, circMAN1A2 binds to SFPQ protein, which plays a significant role in tumorigenesis and progression, and prevents protein degradation. Furthermore, exosomes released by GC cells were found to significantly trigger cell cycle arrest in CD8+ T cells, altering their gene expression and cytokine secretion levels [40].

Cancer cell-derived exosomes may also carry a type of regulatory non-coding RNA, microRNA, which acts as a signal modulator that participates in intercellular communication. Thus, nasopharyngeal carcinoma (NPC) cells were shown to release exosomes with upregulated miRNAs, including miR-24-3p, miR-891a, miR-106a-5p, miR-20a-5p, and miR-1908 [26]. Tumor-secreted exosomes upregulated proinflammatory cytokine levels, such as IL-2, IL-6, IL-10, and IFN-γ, resulting in the creation of metastatic niche in the distant organs through processes like angiogenesis, extracellular remodeling, and immune cell recruitment [65]. In addition, exosome-incorporated miRNA down-regulated the MARK1 signaling pathway and altered T cell proliferation and differentiation.

### 2.2. Modulation of Macrophages

Another major player in the innate immune system is macrophages. These immune cells are involved in various complex processes, including tissue homeostasis, fighting infections, and clearing cellular debris [66]. To execute those different functions, macrophages have unparalleled plasticity that allows them to react to tissue-specific signals and change their actions. The adaptability of macrophages is key for keeping physiological balance; however, in pathological conditions, this feature is exploited by cancer cells manipulating their diverse molecular programs [67]. Thus, reprogramming occurs not only to tissue residential macrophages, but also to monocytes recruited into tumor sites via disease-associated signals. These monocytes impact tumor growth and progression; as a result, they differentiate into macrophages with altered functions, called tumor-associated macrophages (TAMs) [68]. Specifically, TAMs play diverse roles in controlling tumor colonization and progression by influencing tumor invasiveness, modulating local immune responses, and promoting angiogenesis. Importantly, these signaling molecules are often incorporated into exosomes released from TME and cancer cells. Cancer cell-derived exosomes can contain various molecules such as growth factors and miRNAs that have different effects on the target cells of the TME.

Lewis lung carcinoma (LLC) cells release exosomes with overexpressed tripartite motif-containing 59 (TRIM59) [35]. It was demonstrated that TRIM59 incorporated into exosomes was efficiently transferred to and internalized by tumor-associated macrophages. Accumulated in macrophages, exosome-bound TRIM59 interacts with a lipolytic co-activator, ABHD5, and regulates ABHD5 through proteasomal degradation by ubiquitination. The decline in ABHD5 induces metabolic reprogramming, enabling activation of the NLRP3 inflammasome signaling pathway and secretion of proinflammatory cytokine IL-1β. This activation contributes to immune suppression, vascular permeability, and extracellular matrix remodeling, all of which enhance tumor progression [69]. As a result, the activation of macrophages by TRIM59-containing exosomes promotes proliferation, migration, and invasion of lung cancer cells.

Macrophages are generally classified based on their polarization status into classically activated (M1) and alternatively activated (M2) types. In tumor tissues, M1 macrophages are known for their tumor-killing properties, while M2 macrophages promote tumor growth by supporting angiogenesis and metastasis. The mechanism of transformation of antitumor-activated M1 macrophages into M2-polarized TAM by cancer cells was investigated by Chia-Hsin Hsieh et al. [25]. It was demonstrated that human head and neck cancer cells release exosomes with incorporated microRNA-21 (miR-21) which are efficiently accumulated by CD14+ human monocytes. The delivery of miR-21 resulted in the suppression of M1 markers and the increasing expression of M2 markers. Tumor suppressor genes, such as PTEN and PDCD4, are downregulated by miR-21, which inhibits proinflammatory cytokines in addition to the upregulation of M2 markers [70]. Importantly, the epithelial–mesenchymal transition (EMT) transcriptional factor Snail directly activated the transcription of miR-21 in cancer cells and its packaging into exosomes. Notably, when miR-21 was knocked down in the cancer cells, the polarization of macrophages into M2 type was attenuated. Moreover, Snail-expressing cancer cells release several cytokines (CCL2, CCL5, and IL-8) that enhance the recruitment of macrophages into tumor tissues [71]. Conversely, it was reported that paired-like homeodomain transcription factor 1 (PITX1) packaged into exosomes released from osteosarcoma (OS) cancer cells mediates M2 macrophage polarization to promote OS metastasis via CCL22 [72].

The modulating effects of cancer-released exosomes on macrophages appeared to be a universal feature for many types of tumors. For example, bladder cancer cells secrete exosomes with miR-21 and can modify THP-1 cell-derived macrophages into the M2 phenotype [31]. The mechanism of this polarization by exosomal miR-21 is related to the inhibition of phosphatases, along with activation of the PI3K/AKT signaling pathway in macrophages. This increased STAT3 expression and promoted phenotypic polarization of TAMs. In STAT3 activation, immunostimulatory molecule production is reduced and their ability to inhibit tumor cell growth is compromised, making it a critical step in TAM induction [73]. Accordingly, exosomes derived from another bladder cancer cell, MB49, induced immunosuppressive polarization of residential macrophages due to down-regulated tumor suppressor gene PTEN and the activation of the AKT/STAT3/6 signaling pathway [32]. Furthermore, significant effects of miRNA-503-positive exosomes released by breast cancer cells were associated with the loss of X-inactive specific transcript, XIST [74]. Interestingly, the downregulations of XIST expression levels affected the development of brain metastasis but not bone metastases. It was found that loss of XIST altered the secretion of miRNA-503 incorporated into exosomes, which promoted M1-M2 polarization of microglia. This M1-M2 conversion also resulted in the release of immune suppressive cytokines in microglia that in turn, suppressed T cell proliferation. All these effects promoted brain metastatic growth [74].

### 2.3. Expansion of Myeloid-Derived Suppressor Cells (MDSCs)

MDSCs are a heterogeneous population of cells of myeloid origin that expand in pathological conditions like cancer development. They have a remarkable ability to suppress T cell responses. The mechanisms that drive this suppression include the production of suppressive factors such as arginase, reactive oxygen species, and inducible nitric oxide synthase which are all key components of the TME [75,76]. In addition to their ability to impair T cell receptor signaling and proliferation, the TME is altered to favor tumor progression. It was reported that during tumorigenesis, cancer cells release exosomes that affect the development of MDSCs, facilitating their activation, promoting their expansion, and enhancing their immunosuppressive functions [77]. The tumorigenic effects of activated MDSCs include increased proliferation, angiogenesis, migration, and immune escape by cancer cells [78]. Thus, cancer-associated exosomes initiate regulatory mechanisms responsible for the modulation of functional MDSC induction. Furthermore, exosomes released from mammary carcinoma carry abundant PGE2 and TGF-β molecules that increase the expansion and immunosuppression of MDSCs. This process occurs due to the increased production of IL-6 via STAT3 signaling and VEGF, which drives angiogenesis and recruits additional immunosuppressive cells into the TME [36,79,80,81].

Next, tumor cells, including colon carcinoma, mammary carcinoma, and lymphoma, release exosomes with heat shock protein Hsp72 expressed on their membrane. In response to stress or damage, membrane-bound Hsp72 acts as an activator of intracellular signaling pathways [37,82,83]. Delivered to MDSCs, exosome-incorporated Hsp72 triggers STAT3 activation and production of IL-6. This results in a potent inhibition of immune surveillance and promotion of MDSC suppressive functions. Correspondingly, exosomes from multiple myeloma cells deliver IL-10 and IL-16 to MDSCs and activate the STAT3 pathway, suppressing antitumor response [84]. The delivery of immunoregulatory cytokines enhances the proficiency of MDSCs, allowing tumors to evade immune destruction.

Along with bioactive proteins, cancer-derived exosomes deliver miRNAs to MDSCs. Thus, exosomes released from glioma and lung cancer cells were shown to carry high levels of several miRNAs that are involved in regulating multiple cellular processes. These miRNAs include miR-126-3p, miR-27b, miR-320, miR-342-3p, miR-29a, and miR-92a [85,86,87]. These exosome-incorporated mRNAs stimulate the differentiation of functional MDSCs and their propagation.

Next, exosomes released from hypoxia-induced glioma were shown to carry miRNA-21 and miR-10a that promote the expansion and immunosuppression of MDSCs by targeting PTEN and RORα [33]. Accordingly, exosomes released from oral squamous cell carcinoma were reported to deliver miRNA-21 and miR-10a and affect MDSCs in an miR-21/PTEN/PD-L1 axis-dependent manner [88]. This signaling axis promotes the expansion of MDSCs and upregulates PD-L1 expression, which suppresses T cell function and contributes to tumor immune escape [89]. Furthermore, miR-1260a and miR-494-3p were delivered to MDSCs by exosomes released from B-cell-derived chronic lymphocytic leukemia [34]. Accumulation of these exosomes led to increases in calcium fluxes through the transfer of SMAD4-related, differentially expressed miR-1260a and miR-494-3p. Finally, high levels of miRNA-155 in chronic lymphocytic leukemia-derived exosomes caused immunosuppressive effects through the STAT1 pathway [34].

### 2.4. Toxic Effects on Dendritic Cells

**Figure 1:**
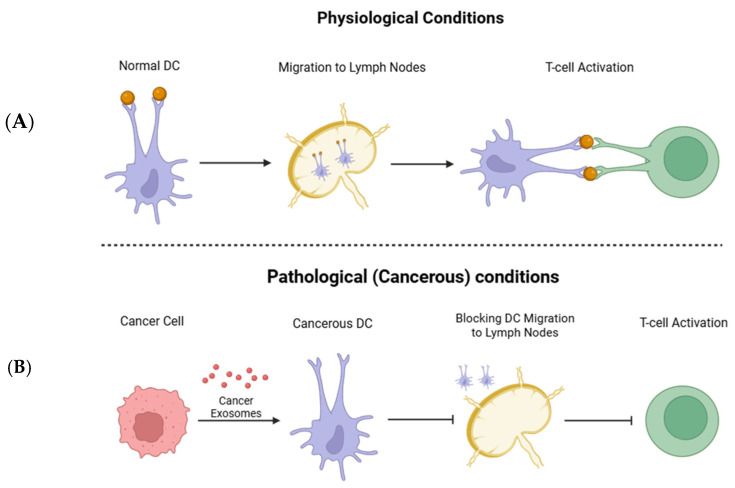
Role of dendritic cells (DCs) in antitumor immunity and their suppression by cancer-derived exosomes. (**A**) Under normal physiological conditions, DCs capture tumor antigens, migrate to lymph nodes, and activate T cells, thereby initiating an effective antitumor immune response. (**B**) In the presence of cancer-derived exosomes, DC migration to lymph nodes is inhibited and T cell activation is suppressed, promoting immune evasion.

One of the active participants in the TME is antigen-presenting cells, i.e., dendritic cells (DCs). These cells play a significant role in the defense against tumor development. DCs recognize and present tumor antigens to T cells, initiating and modulating immune responses against cancer. Antigen presentation is necessary for activating cytotoxic T-lymphocytes to target and eliminate tumor cells. Consequently, T cell elimination or inactivation would promote cancer growth and development. In this respect, cancer cell-released exosomes were reported to inhibit the chemokine receptor expression in DCs, and as a result, block their migration to secondary lymphoid organs [38]. Impaired DC migration prevents these cells from reaching the lymph nodes where they would prime naive T cells (Figure 1). Overall, it leads to a weakened immune response due to the inability to initiate an adaptive immune response [90,91]. Thus, exosomes released from Lewis lung carcinoma (LLC) or 4T1 breast cancer cells were shown to prevent the differentiation of myeloid precursor cells into DCs and promote cell apoptosis. The authors suggested that this exosome-induced immune suppression is related to the overexpression of PD-L1. Exosome-incorporated PD-L1 can bind to PD-1 receptors on immune cells, such as DCs and T cells. These cells transmit inhibitory signals to dampen immune activation and promote immune tolerance [19,92]. Correspondingly, human colorectal carcinoma and melanoma cells were shown to release exosomes that inhibit the differentiation of human monocyte precursors to DC [93].

Finally, it was demonstrated that cancer-derived exosomes can deliver maturation-inducing signals and induce distinct phenotypic changes in DC, switching the maturation profile and inducing significant death of DC [39]. Of note, this effect was observed for exosomes released by different mouse tumor cell lines, including B16F10, MCA101, LLC1, KP, and EO771.

### 2.5. Reprogramming of Fibroblasts

Cancer–stroma crosstalk can also be facilitated by cancer-derived exosomes. As part of the body’s natural defense, at the early stages of cancer development, fibroblasts release signaling molecules that inhibit cancer growth. To restrict uncontrolled cell growth, tumor-suppressive cytokines and growth inhibitory factors prevent malignant transformation of cells [94]. Later, they help repair tissues and maintain normal structure, which can prevent cancer formation. To overcome this process, cancer cells release exosomes that suppress fibroblast functions and provide favorable conditions for tumor development. Specifically, the exosomes released by cancer cells promote the transformation of normal fibroblasts into tumor-associated fibroblasts (TAFs). This transformation reprograms fibroblasts to allow cancer progression and enhanced metastasis [41,95].

Specifically, various miRNAs in cancer cell-released exosomes play an important role in reprogramming normal fibroblasts into TAFs. Thus, studies indicate that exosomes released from breast cancer cells carrying a prometastatic miRNA, miR-9, modify human breast fibroblasts and transfer them into TAFs [42]. This effect resulted in enhanced cell motility and promotion of tumor growth. Furthermore, miR-200-positive exosomes released from breast cancer cells activate TAFs and increase cancer cell invasion [96]. In addition, the reprogrammed normal fibroblasts showed downregulated miR-31 and miR-214, and upregulated miR-155 [97]. Thus, ovarian cancer cells modified normal fibroblasts to become TAFs through the act of miRNAs incorporating into exosomes, which confirms that tumor cells can manipulate the stromal environment to promote cancer progression.

Moreover, exosomes derived from TP53-deficient colon cancer cells were reported to carry several microRNAs (miR-1249-5p, miR-6737-5p, and miR 6819-5p) that were accumulated in TAFs [43,98]. It was revealed that these exosomes transfer normal fibroblasts into TAFs and promote tumor development. Furthermore, it was demonstrated that cancer-associated fibroblasts can accelerate the proliferation of cancer cells by secretion of diverse signal molecules, including growth factors (i.e., stromal cell-derived factor 1 (SDF-1)), chemokines, and cytokines (TGF-β or VEGF) [99,100]. These signaling molecules are responsible for the enhancement of cancer cell migration, invasion, and other aspects of tumor progression in addition to suppressing immune responses [101].

### 2.6. B Cell Transformation

**Figure 2:**
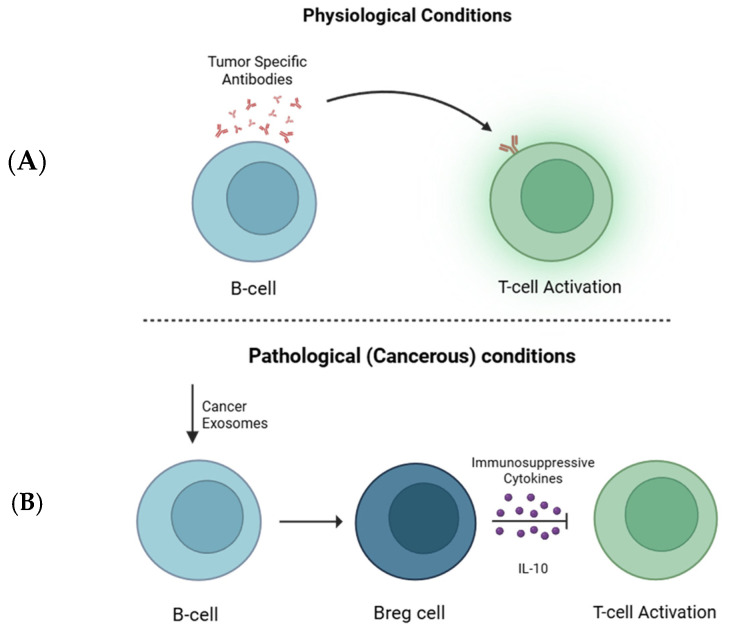
B cell roles in antitumor immunity versus tumor-induced immunosuppression. (**A**) Under normal conditions, B cells recognize tumor-specific antigens and produce antibodies to activate T cells, initiating an adaptive immune response. (**B**) Cancer-derived exosomes transform B cells into regulatory B cells (Bregs), which secrete immunosuppressive cytokines such as IL-10, suppressing T cell activation and promoting tumor immune evasion.

Although most of the research on the immune response to tumor development focuses on T cells and macrophages, B lymphocytes also play a key role in this process. Under normal physiological conditions, B cells produce tumor-reactive antibodies and activate other immune cells, such as T cells and NK cells, as shown in Figure 2A. B cells produce immunoglobulin (Ig) and secrete proinflammatory cytokines. Thus, it was reported that cancer-derived exosomes with overexpressed T cell Ig and mucin domain (TIM)-1 protein activate B cells and promote their suppressive activity against CD8+ T cells [44]. However, as a tumor grows, cancer cells release exosomes with signaling molecules that modify B cells into a specific pro-tumorigenic phenotype known as regulatory B cells (Bregs). One mechanism, by which Breg suppresses the function of T cells is through the high expression of the immunosuppressive cytokine IL-10 (Figure 2B). IL-10 inhibits T cell activation by dampening its function and promoting immune tolerance in the TME [102]. In line with this, the expansion of TIM-1+Breg cells was promoted through the Toll-like receptor (TLR) 2/4 and mitogen-activated protein kinase (MAPK) signaling pathways.

### 2.7. Suppression of NK Cell Activity

Being a type of lymphocyte, NK cells play an important role in the immune system by eliminating cancer cells. NK cells work rapidly and can detect and eliminate abnormal cells early on without needing sensitization from the adaptive immune system first. To evade their actions, cancer cells release exosomes that downregulate surface expression of the activating receptor NKG2D in NK cells and impair cell cytotoxic functions [23,45].

These exosomes can alter the phenotype of NK cells and carry immunosuppressive molecules in addition to the soluble ligands for NKG2D [103]. They also carry growth factors, such as membrane-associated transforming growth factor-β1 (TGFβ1) and weaken the capacity of NK cells to recognize and destroy cancer cells. Thus, TGFβ1-loaded exosomes play a principal role in NK cell inactivation. Affected by cancer-derived exosomes, these NK cells showed low production of interferon gamma (IFN-γ) and poor NKG2D-dependent killing function. Likewise, exosomes collected from patients with acute myeloid leukemia also contained high levels of TGFβ1 [46]. Correspondingly, these exosomes were shown to decrease NK cell cytotoxicity and downregulate the expression of NKG2D in normal NK cells.

### 2.8. Effect on Tumor-Associated Neutrophils

Neutrophils also play a crucial role in both innate and adaptive immunity, fighting infection. These cells act as the body’s primary defense, as they rapidly respond to foreign bodies in a host by engulfing them. Neutrophils act as mediators in initiating an immune response, regulating inflammation, and repairing tissues [104]. In pathological conditions, they accumulate at the diseased tissues and shape the host response to infection and immune system homeostasis [105]. Similarly to other immune cells, neutrophils interact with cancer cells through their messengers, exosomes, and vice versa. It was reported that cancer cell-derived exosomes interact with tumor-associated neutrophils (TANs), causing the formation of neutrophil extracellular traps (NETs), which contribute to the prothrombotic phenotype in cancer [47,106,107]. Specifically, exosomes released from 4T1 murine mammary carcinoma cells caused accelerated thrombus formation in BALB/c mice, suggesting that these cancer cells promote vascular complications and facilitate metastatic spread. It was shown that NETs may serve as a scaffold for tumor-derived procoagulant exosomes. The importance of tissue factor (TF) carried by cancer-derived exosomes was proposed [108].

### 2.9. Effect on Neighboring Cancer Cells

Exosomes released from cancer cells not only impact the TME, but also modulate neighboring cancer cell growth in an autocrine manner [48,49,109,110]. For instance, McCready et al. demonstrated that in both breast cancer and glioma cell lines, cancer-cell-derived exosomes containing surface HSP90α were shown to increase migration and proliferation on recipient glioma and breast cancer cells [48]. In 2016, Sakha et al. demonstrated that miR-342-3p and miR-1246 were upregulated in exosomes released by a highly metastatic human oral cancer cell line, and these exosomes increased motility and invasive ability of the recipient oral cancer cell line that was poorly metastatic [49]. Similarly, RAB27A was found to increase invasion in melanoma cell line [110]. Taken together, exosomes released from cancer cells can promote cancer progression through promoting proliferation and migration in recipient and neighboring cancer cells.

## 3. Crosstalk of TME-Released Exosomes with Cancer Cells

Apart from tumor cells, exosomes from the TME also contribute to tumor development. In fact, the TME has a major function in tumor progression and metastasis. As discussed above, cancer-released exosomes target the TME to reduce the anticancer response, creating an immunosuppressive, protumor surrounding. On the other hand, exosomes released by nontumor cells in the TME may also affect cancer cells through the transfer of regulatory biomolecules (summarized in Table 2). This enhances tumor cell behavior by leading to tumor growth, invasion, metastasis, and immunotherapeutic resistance. In this section we will discuss how TME-released exosomes promote cancer development.

### 3.1. Activation of Tumor Cell Proliferation and Growth

TME cells use different effector molecules packed into exosomes to induce and promote growth of cancer cells. For example, exosomes released by M2 macrophages can deliver the antisense of leucine-rich repeat-containing protein 75A (LRRC75A-AS1) to HeLa cells, inducing cervical cancer progression [111]. This protein targets and suppresses miR-429, an microRNA that normally functions to inhibit SIX1 expression, to suppress the invasion and migration of cervical cancer cells. By downregulating miR-429, exosome-incorporated LRRC75A-AS1 demonstrated upregulation of SIX1 expression, a key transcription factor that drives the EMT, and subsequently induced Hela cell functions through STAT3/MMP-9 signaling. Furthermore, the activation of STAT3/MMP-9 signaling furthers tumor progression and dissemination by enhancing extracellular matrix degradation. Exosomes from tumor-associated macrophages (TAMs) can also deliver miR-589-3p that promote ovarian cancer (OC) development [112,121]. miR-589-3p is a multifunctional miRNA involved in a wide range of biological activities, including tumor proliferation or metastasis. This miRNA functions as a potent oncogenic regulator, targeting BCL 2-like protein 13 (BCL2L13), which is involved in promoting apoptosis through mitochondrial pathways [122,123]. The disruption of BCL2L13 expression by miR-589-3p weakens the cell’s natural apoptotic processes, which in turn promotes tumor survival. The exosomal transport of miR-589-3p enables its effective transfer throughout the tumor microenvironment, enhancing communication between TAMs and OC cells. It was demonstrated that miR-589-3p delivered by TAM-derived exosomes can suppress expression of BCL2L13, enhance OC cell proliferation, and suppress OC cell apoptosis [111]. Next, Chen et al. reported that exosomes released by TAMs deliver circular RNAs (CircRNAs), specifically Circ_0020256, that promoted the proliferation, migration, and invasion of cholangiocarcinoma (CCA) cells [124]. It was demonstrated that Circ_0020256 acts as a competing endogenous RNA (ceRNA) and interacts with its intracellular microRNA target, miR-432-5p. By binding to miR-432-5p, Circ_0020256 prevents the tumor-suppressive miRNA from inhibiting its downstream targets. This suppresses the oncogenic pathways, thereby creating a more aggressive tumor behavior [125]. Of note, treatment with small interference RNA (siRNA) for Circ_0020256 inhibited CCA cell proliferation, migration, and invasion both in vitro and in vivo.

Finally, exosomes released by neural progenitor cells (NPCs) with significantly overexpressed miR-99a-5p affect neighboring cancer cells and facilitate their proliferation and migration through targeting the BAZ2A gene, which plays a crucial role in regulation of non-coding RNAs and chromatin remodeling [126]. miR-99a-5p downregulates BAZ2A, thereby disrupting normal epigenetic regulation, and activating tumor-promoting genes involved in tumor growth and mobility. Through exosomal delivery, miR-99a-5p is delivered to neighboring cancer cells. This amplifies the tumorigenic effects of NPCs within the TME. In addition, oncogenic miRNAs such as miR-21, miR-141, and miR-451 were reported to increase proliferation and invasion of cancer cells by affecting pathways involved in apoptosis, invasion, and matrix degradation [127,128]. These miRNAs function by regulating targets such as PTEN, MMPs, and TIMP3, allowing cancer cells to evade apoptosis, alter the extracellular matrix, and become more invasive.

### 3.2. Escaping Immune Response

Immunocyte-released exosomes deliver their biologically active compounds not only to cancer cells, but also to other immune cells. Thus, it was reported that exosomes released by exhausted CD8+ T cells can enter normal CD8+ T cells and inhibit their proliferation and cell activity. The exosomal transfer of dysfunctional signaling molecules creates a negative feedback loop that further compromises the immune response against tumors by enhancing T cell exhaustion [129]. They also decrease production of cytokines, such as interferon-γ and interleukin-2 by normal T cells [130]. Furthermore, immunosuppressive M2 macrophages release exosomes that render cancer cells resistant to CD8 T cells [118]. These exosomes deliver apolipoprotein E (ApoE) to cancer cells that make them undetectable by downregulation of MHC-I expression and inhibition of tumor-intrinsic immunogenicity. To avoid detection by cytotoxic lymphocytes, cancer cells disrupt antigen presentation to enable uncontrolled growth. The mechanism involves decreased tumor-intrinsic ATPase activity that results in the inhibition of binding immunoglobulin protein (BiP) and decrease in MHC-I expression by cancer cells. Diminished BiP function disrupts protein folding and stability in the endoplasmic reticulum, leading to a loss of surface MHC-I expression and reduced immune recognition. Next, immunocyte-derived exosomes that circulate in the blood of cancer patients were reported to carry PD-1 and CD80 on their membranes, contributing to systemic immunosuppression and tumor progression by regulating immune signaling and modulating the function of the recipient cells upon uptake of the vesicle [131,132]. Thus, PD-1/CD80+ exosomes convert tumors to an immunologically cold phenotype through adaptive redistribution of PD-L1 in tumor cells, enabling tumor cells to shift PD-L1 expression to immune interaction sites, and thereby dampening cytotoxic T cell activation and promoting immune escape. Interestingly, a simultaneous increase in PD-1 and CD80 on the surface of T cell-derived exosomes was revealed in cancer patients. Of note, a recent study suggests that co-expression of CD80 in exosomes carrying PD-L1 results in inhibition of immunosuppressive and tumor-promotive effects [10]. For example, several immune cells, including T cell, B cell, dendritic cell, and monocyte/macrophage were shown to release PD-L1-positive exosomes. It was demonstrated that CD80 interferes with the binding of PD-L1 to its receptor PD-1 on T cells and therefore restricts immunosuppression.

Furthermore, exosomes released from M2-TAMs were shown to be rich in LINC01592, which is a type of LncRNA that exhibits a longer sequence length and a more intricate spatial structure in contrast to small-molecule RNA. This complex structure of LINC01592 enables it to interact with various cellular proteins, affecting both transcriptional and post-transcriptional regulation. These exosomes carry LINC01592 into esophageal cancer (EC) cells, where LINC01592 directly binds to E2F6 and promotes E2F6 entry into the nucleus [114]. Consequently, the two synergistically promote the transcriptional level of NBR1 and the increased binding of NBR1 to the ubiquitinated protein MHC-I. In addition, LINC01592 mediated nuclear localization of E2F6, resulting in the suppression of WISP1, which is a recognized inhibitor of the EMT. Therefore, reduced WISP1 expression promotes EMT, increasing tumor cell invasion and metastasis. Consequently, exosomes enriched with LINC01592 support both immune evasion and the aggressive advancement of esophageal cancer. This leads to the degradation of MHC-I in auto-phagolysosomes and its decreased expression on the surface of tumor cells, which in turn leads to tumor cells escaping immune attack. Ultimately, LINC01592-enriched exosomes facilitated immune evasion of EC cells by avoiding CD8+ CTL cells, contributing to resistance against immunotherapies.

### 3.3. Promoting Metastases

TME-derived exosomes promote metastasis not only through cancer cell proliferation, but also through improved migration. For example, Wang et al. reported that exosomes carrying miR-223-3p promote development of pulmonary metastasis of breast cancer 4T1 cells [115]. Specifically, exosome-bound miR-223-3p targets chromobox 5 (Cbx5), which has been widely reported as a suppressor of cell migration and invasion [133,134]. Cbx5 is an essential part of chromatin remodeling complexes, regulating gene expression involved in maintaining cell adhesion and restricting cell movement, thus acting as a metastasis suppressor. Moreover, miR-223-3p was confirmed to increase the migration and metastasis of 4T1 cells, affecting their motility. As a result, miR-223-3p in TAM-derived exosomes enhances lung metastasis of 4T1 cells via Cbx5. Furthermore, exosomes released by TAMs deliver functional Apolipoprotein E to gastric cancer cells, and promote their migration through activating the PI3K-Akt signaling pathway [116].

### 3.4. Promoting Drug Resistance in Cancer Cells

Increasing evidence confirms that exosomes released by TAMs may promote drug resistance in cancer cells. For example, miR-21-positive exosomes released by M2 macrophages transfer their content to gastric cancer cells and promote their resistance to cisplatin, a primary chemotherapeutic drug for gastric cancer patients [117]. miR-21 delivered by TAM-derived exosomes downregulates PTEN, leading to a more active signaling through the PI3K/AKT pathway. In addition, TAM-released exosomes protect cancer cells from cisplatin-induced apoptosis through the regulation of anti-apoptotic protein Bcl-2. Interestingly, this effect is not due to the upregulation of ABC transporter genes but is related to a decrease in chemosensitivity and inhibition of apoptosis in gastric cancer cells, highlighting the complex role of TAM-derived exosomes in mediating chemoresistance within intracellular signaling.

Furthermore, exosomes released by cancer-associated fibroblasts (CAFs) were shown to promote drug resistance in pancreatic adenocarcinomas (PDACs) [113,119]. There are multiple mechanisms that exhibit this effect. First, CAFs are innately resistant to gemcitabine, so when CAFs-derived exosomes accumulate in cancer cells, they induce cells resistant to this nucleoside analog, which is the standard chemotherapeutic agent. Second, when CAFs are exposed to gemcitabine, the number of released exosomes greatly increase. It was found that these exosomes contain a chemoresistance-inducing factor, Snail (SNAI1) as well as the Snail target, microRNA-146a, and that in recipient epithelial cells, they can promote proliferation and drug resistance. Both of these components are known to drive EMT and modulate gene expression toward a migratory and drug-resistant phenotype. Similarly, TAF-derived exosomes were shown to inhibit apelin receptor (APJ) expression [135] which caused TP53 deficiency in the human colon cancer cell line, HCT116 [136]. The decrease in APJ levels resulted from the interaction of fibroblasts with microRNA 5703 incorporated into exosomes derived from cancer cells. This results in accelerated tumor growth.

The induction of chemotherapy and radiation resistance in breast cancer cells was demonstrated by exosomes released from stromal cells [120]. The miRNA in exosomes stimulates the pattern recognition receptor RIG-I and activates STAT1-dependent signaling, strengthening the DNA repair processes and supporting cell survival when exposed to cytotoxic stress. In addition, exosomes from stromal cells also activate NOTCH3 on breast cancer cells, inducing resistance to anticancer therapy. Of note, the NOTCH family of receptors is responsible for the activation of developmental signaling pathways that promote drug resistance through its role in sustaining cancer stem cell characteristics, preserving tumor cell adaptability and fostering self-renewal [137].

### 3.5. Antitumor Activities

The physiological function of immune cells includes anticancer activity. Although cancer cells can modulate the TME to promote conditions for cancer cell proliferation, exosomes within the TME can also regulate cancerous cells. For instance, several studies have demonstrated that exosomes released by tumor or stromal cells can transport tumor antigens to dendritic cells, facilitating their presentation via MHC-I molecules, inciting an antitumor immune response [138,139]. Additionally, M1 macrophages in the TME secrete exosomes containing proinflammatory cytokines such as TNF-α and IL-12 which enhance T cell activation and inhibit tumor progression [140].

## 4. Targeting Exosomes as a Potent Therapeutic Option for Cancer Treatment

Understanding the cellular and molecular underpinnings of exosomes released from cancer cells is a prerequisite for the development of successful treatment approaches for this deadly malignancy. Exosomes play a critical role in shaping this interplay, and disrupting these detrimental connections may provide new and powerful solutions for cancer treatment, given their capacity to carry bioactive molecules that serve as therapeutic targets. Comprehensive knowledge of exosome-mediated tumor growth helps to develop therapeutic strategies and innovative treatments. For instance, cancer-derived exosomes are a potential therapeutic target to prevent immunosuppression and enhance antitumor immune responses. Immunotherapy in the context of anticancer treatment has emerged as a prominent therapeutic approach. Much effort has been dedicated to the development of strategies that aimed at the suppression of exosome release from cancer cells and TME. Importantly, in many cases, effects on exosome formation and release occurred through multiple mechanisms including inhibition of metastases development, activation of immune response, and reduction in drug resistance via higher accumulation of anticancer drugs in tumors. Here we discuss several therapeutic approaches targeting cancer exosomes.

### 4.1. Inhibition of Formation and Release of Cancer Exosomes

Exosome inhibition can be achieved via multiple strategies that affect different mechanisms. Several pharmacological agents were investigated as inhibitors of exosome release to attenuate cancer growth. Thus, compounds such as pantethine, imipramine, simvastatin, cannabidiol, sulfisoxazole, and macitentan were shown to suppress exosome production and release [141,142,143,144,145,146,147]. The inhibition of the endosomal sorting complex required for transport (ESCRT) machinery is a powerful tool to disrupt cancer exosome production. Thus, sulfisoxazole (SFX), an oral antibiotic, was reported to inhibit the secretion of exosomes from breast cancer cells [147]. It was demonstrated that SFX can suppress the components of the ESCRT pathway, including ALIX and VPS4B. In addition, SFX disrupted the transcription of Rab GTPases in cancer cells. As a result, significant antitumor and antimetastatic effects were observed in mice with breast cancer xenografts. In another study, heparan sulfate (HS) analogs were used to inhibit cancer exosome release through the targeting of the syndecan–syntenin–ALIX complex [148]. Furthermore, disruption of ESCRT-independent pathways can also play a significant role against formation and release of cancer exosomes. For example, the vitamin B5 derivative, pantethine, and the calpain inhibitor, PD-150606, were shown to inhibit cancer-derived exosomes, and as a result, attenuates tumor growth [141]. Specifically, it was reported that pantethine is involved in the regulation of lipid and cholesterol metabolism [149]. The inhibition of lipid and cholesterol synthesis resulted in a decrease in exosome production. Interestingly, along with the inhibition of cancer exosome release, pantethine also reduced multidrug resistance of cancer cells, suggesting a potential role for calcium in their mechanism of action. Another potent anticancer agent, GW4869, is a noncompetitive inhibitor of the neutral sphingomyelinase-2, nSMase2, which is an enzyme required for exosome release [143]. It was reported that GW4869 significantly suppressed tumor growth and increased murine survival in mice injected subcutaneously with B16BL6 cells [144]. GW4869 was shown to efficiently block exosome generation and reduce exosome release in cancer cells. Of note, this process did not depend on the function of ESCRT machinery. Furthermore, imipramine, originally used as an antidepressant, was also shown to inhibit exosome biogenesis through the inhibition of acid sphingomyelinase [145,149]. Similarly, simvastatin, an HMG-CoA reductase inhibitor, has been reported to significantly reduce exosome secretion in both epithelial cells and monocyte [150]. Furthermore, cannabidiol was found to inhibit exosome release by 50% in cancer cells, including PC3 liver cancer cells, HEPG2 breast cancer cells, and MDA-MB231 cells [146]. The mechanism of exosome inhibition is related to interference with CD63 expression in cancer cells. In another study, tipifarnib, a FTase inhibitor, was used to inhibit exosome secretion from the prostate cancer cells [151,152]. Finally, epidermal growth factor (EGF) was used to block the release of cancer exosomes in oral squamous cell carcinoma (OSCC) [153]. This resulted in several effects including decrease in proliferation, migration, invasion, and chemoresistance of OSCC cells. The inhibition of exosome production may also be achieved by affecting the acidic microenvironmental pH that regulates vesicular trafficking in cancer cells. An acidic pH within the TME promotes the formation and release of exosomes, thereby facilitating tumor growth and contributing to drug resistance. Thus, proton pump inhibitors of vacuolar-type (V-type) H(+)-ATPases were used to inhibit exosome release [154,155]. For example, a proton pump inhibitor lansoprazole caused a significant reduction in exosome release from human melanoma cells [156]. This resulted in marked increase in the uptake of the anticancer drug, cisplatin, and inhibition of tumor growth. Moreover, regulation of intracellular signaling pathways, and, in particular, lysosomal trafficking was achieved using the regulator complex subunit, LAMTOR1, which facilitates autophagy by limiting its interaction with vacuolar H+-ATPase [157]. This accelerated PD-L1 degradation in lysosomes, thereby improving immune response to the tumor. It was reported that treatment with LAMTOR1 suppressed the release of cancer exosomes carrying PD-L1 and promoted CD8+ T cell infiltration and cytotoxic activity in non-small cell lung cancer (NSCLC) [155]. Thus, using peptides that target PD-L1 for lysosomal degradation can significantly improve the anti-PD-1 immunotherapy.

### 4.2. Disrupting Cancer Exosome–Cell Interactions

To enter target cells, exosomes utilize specific receptors that are located on their surface. To inhibit this mechanism, different antibodies against CD9 and CD63 were proposed. Thus, it was shown that treatment with human-specific anti-CD9 or anti-CD63 antibodies can considerably reduce the development of metastasis in the lungs, lymph nodes, and thoracic cavity, in mice with xenograft tumors [158]. However, this treatment can be significantly diminished due to antibodies’ clearance by macrophages in the circulation. Furthermore, M. Santos et al. reported the successful employing of anti-human CD9 Fab fragment antibody to intercept exosome-based intercellular communications [159]. The anticancer potential of anti-CD9 Fab antibody was demonstrated with highly metastatic SW620 cells. Treatment with anti-CD9 Fab antibody altered the mesenchymal-like morphology of cancer cells and inhibited their migration. In another work, researchers suggested blocking tetraspanin CD9-mediated signaling on isolated exosomes that impaired migration of pancreatic ductal adenocarcinoma in cells [160]. Similar results were obtained by T. Suwatthanarak et al., in which it was demonstrated that an eight-mer CD9-binding peptide inhibited secretion of cancer exosomes, decreased cancer cell migration, and inhibited metastases development in a mouse model of lung cancer [161]. Moreover, blocking exosomal integrins, such as α6β4 in lungs or αv β5 in liver, using antibodies against these receptors was also shown to reduce development of metastases [162]. Interestingly, it was demonstrated that expression of these integrins on the surface of cancer-related exosomes is required to achieve specific tropism to these organs. This provides rationale for using antibodies against integrin-mediated docking of cancer-derived exosomes. Finally, heparan sulfate proteoglycans (HSGs) can be used as internalizing receptors for cancer-derived exosomes [163]. Inhibition of this mechanism, for example, by oligosaccharide blockade, may play a significant role in reducing cancer metastases. Further investigations revealed that blocking of exosome uptake occurred at the level of cell binding and not internalization [164]. Obviously, heparin by itself cannot be used as an antimetastatic therapeutic due to the bleeding risk. However, heparin mimetics could be employed for inhibition of cancer-derived exosome internalization. Specifically, small hydrophobic compounds, xylosides, were tested for pharmacological inhibition of exosome-dependent cell migration [163]. It was shown that treatment with 4-nitrophenyl β-d-xylopyranoside resulted in approximately 50% inhibition of exosome uptake in target cells. Another case with monoclonal antibodies, anti-cytoskeleton-associated protein 4 (CKAP4), was reported for treatment of pancreatic ductal adenocarcinoma (PDAC) [165]. The secreted exosomes showed low abilities to bind to DKK1 and CKAP4, resulting in decreases in proliferation and migration of PDAC cells.

### 4.3. Elimination of Cancer Exosomes from the Bloodstream

Removal of cancer exosomes from the entire circulatory system is an alternative therapeutic approach [166]. For example, a high amount of mannose structures on the surface of cancer-derived exosomes allows their binding to lectins, including GNA, and elimination from the blood [167]. It was reported that six of the sixteen patients with metastatic cancer had a reduction in the sizes of their tumors by 50% or more. Furthermore, 22 patients out of 144 showed a measurable reduction in tumor burden followed by therapeutic apheresis [168]. This strategy has several advantages compared to chemotherapy, including absence of drug toxic effects and reduced interactions risks. In addition, caner exosomes can be separated using an antibody-based approach. Thus, anti-HER2 antibodies can be utilized to remove HER2-expressing exosomes in patients with HER2 over-expressing breast cancer [169]. This method allows for capturing cancer exosomes while sparing exosomes produced by non-malignant cells. Another potential outcome from this strategy of exosome clearance is early identification, prognosis, and optimization of antitumor treatments. For example, the presence of exosomes with high expression of the hub gene, claudin-7 (CLDN7), which is the marker for triple negative breast cancer (TNBC), may provide valuable information for the clinical diagnosis, treatment, and risk assessment of TNBC [115]. Therefore, cancer exosomes isolated from patients’ blood allow for identification of different cancers using their distinct miRNA profiles [170]. Overall, with advancements in cancer research, increasing attention is being directed toward one of the main strategies: the inhibition of exosome release by cancer cells and TME. These systems could offer a unique strategy for improving the therapeutic outcomes for cancer patients. A considerable amount of the literature indicates that reduction in cancer exosome load causes enhancement of anticancer therapy. However, these approaches still require thorough preclinical evaluation and clinical validation through carefully designed trials to assess their effectiveness and safety before they can be widely adopted in oncology. Several important reviews were conducted concerning using exosomes for drug delivery to treat cancer [171,172,173,174,175,176,177]. In the present review, we were focusing on targeting cancer-derived exosomes as an antitumor strategy.

## 5. Conclusions

Exosomes play a vital role in cell-to-cell communications within a healthy body, and cancer cells exploit these same mechanisms for their own advantage. Generally, exosomes provide targeted, protected, and efficient communication between cells and even between distant organs. First, exosomes provide protection against degradation of biological active compounds, including proteins, lipids, mRNA, miRNA, and even DNA fragments. Many bioactive compounds are unstable in extracellular fluids and in the blood stream. Therefore, when these compounds are incapsulated into exosomes, they become shielded from enzymatic degradation and overall, from the immune cells’ recognition. Furthermore, exosomes are not merely simple membrane-bound vesicles, so exosome-mediated transfer extends beyond passive diffusion. Exosomes are equipped with targeting moieties that enable selective delivery to the target cell or organ. This allows cancer cells to deliver their messages precisely to neighboring cells or distant organs. Finally, cancer cells release exosomes that can reprogram recipient cells. Thus, cancer-derived exosomes can promote metastasis, angiogenesis, and immune evasion.

Today, the high importance of intercellular communications in tumor development and antitumor response and therapy is widely recognized. The heterogeneity of a tumor and the complexity of exosome cargo complicate these investigations. In addition, along with promotion of cancer growth and metastases, tumor-derived exosomes can deliver various tumor antigens that activate anticancer immune responses and therefore, play an anti-tumorigenic role [178]. For example, it was revealed that cancer cells under stress conditions may release proinflammatory cytokines, such as IL-2, or IL12, that enhance anticancer immunity. These complex and sometimes contradicting effects of cancer cell-derived exosomes should be considered. Thus, exosome-mediated reprogramming of immune cells may have different and sometimes opposite effects depending on the nature of the recipient cell. Furthermore, exosomes released by TME can also exhibit a dual role: promoting tumor immune evasion and enhancing antitumor activity. Overall, the advancements in exosome biology pave the way to achieving effective personalized anticancer therapy to improve clinical outcomes. Thus, a deeper understanding of the role of exosomes in these processes and regulatory mechanisms will promote the discovery of new targets for cancer diagnosis and therapy.

## Figures and Tables

**Table 1:** Summary of immune modulation of TME by cancer cell-derived exosomes.

Immune Cell	Upregulated EV Cargo	Effect/Mechanism	Relevant Studies
T cells	PD-L1	Inhibits T cell activation/cytotoxicity via PD-1 pathway, promotes exhaustion, immune evasion	[16,17,18,19,20]
	Fas Ligand (FasL)	Induces apoptosis of activated or CD8+ T cells	[21,22]
	TGF-β, miR-503, miR-21, miR-24	Promotes T cell dysfunction, arrest of activation, exhaustion or suppressor phenotype	[23,24,25,26]
	Tenascin-C	Blocks T cell activation, cytokine release, and supports immune escape	[27,28]
	Mutant p53	Inhibits T cell proliferation and activation	[29]
	TSH/TSHR signaling	Drives T cell exhaustion phenotype, promoting immune evasion	[30]
	Various miRNAs	Directly induce T cell suppression, exhaustion, or apoptosis	[19,24,26]
Macrophages and MDSCs	miR-21, miR-155, miR-10a	Induces M2 (protumor) polarization, activates immunosuppressive phenotype	[25,31,32,33,34]
	TRIM59	Activates NLRP3 inflammasome, supports tumor growth	[35]
	HSP72, TGF-β	Activates STAT3 in myeloid cells, expands/improves MDSC activity	[36,37]
Dendritic Cells	TGF-β, miRNAs	Inhibit maturation, antigen presentation; promote tolerogenic phenotype	[38,39,40]
Fibroblasts	TGF-β, miR-9, miR-21, miR-200	Induce transdifferentiation into CAFs and promote tumor-supportive microenvironment	[41,42,43]
Regulatory B Cells	HMGB1	Expands TIM-1+ regulatory B cells, promotes immunosuppression	[44]
Natural Killer Cells	MICA/B, TGF-β	Downregulates NKG2D, impairs cytotoxicity; TGF-β drives dysfunction	[45,46]
Neutrophils	Tissue Factor (TF), NET components	Induces NET formation, enhances thrombosis, and supports metastasis	[47]
Neighboring Cancer Cells	HSP90α, miR-342-3p, miR-1246	Promotes cancer cell proliferation and migration.	[48,49]

**Table 2:** Summary of EV cargo associated with TME and cancer cell crosstalk.

Donor Cells	Upregulated EV Cargo	Impact on Cancer Cells	Cancer Type	Relevant Studies
M2 and TAM	lncRNA LRRC75A-AS1	Enhances SIX1 expression, promoting proliferation and invasion	Cervical cancer	[111]
	miR-589-3p	Targets BCL2L13 to promote proliferation and metastasis	Ovarian cancer	[112]
	circ_0020256	Activates MEK/ERK signaling, increasing proliferation and migration	Cholangiocarcinoma	[113]
	LINC01592	Decreases MHC-I surface expression, inducing immune escape	Esophageal cancer	[114]
	miR-223-3p	Promotes pulmonary metastasis	Breast cancer	[115]
	Apolipoprotein E	Promotes cancer cell migration	Gastric cancer	[116]
	miR-21	Confers cisplatin resistance	Gastric cancer	[117]
	Immunosuppressive miRNAs	Suppresses tumor immunogenicity and induces immunotherapy resistance	Multiple cancers	[118]
CAF and Stromal	Pro-survival miRNAs and proteins	Enhance survival and proliferation of pancreatic cancer cells	Pancreatic cancer	[119]
	Integrins, regulatory miRNAs, and proteins	Promote metastasis and treatment resistance	Pancreatic cancer	[113]
	RNA and protein cargos activating DNA damage repair pathways	Induce therapy resistance via stromal-to-tumor EV transfer	Breast cancer	[120]

## Data Availability

All data is derived from references cited and found on PubMed.

## Abbreviations

The following abbreviations are used in this manuscript:

EVs	Extracellular vesicles
CNS	Central nervous system
TME	Tumor microenvironment
MDSCs	Myeloid-derived suppressor cells
NK	Natural killer
DCs	Dendritic cells
APCs	Antigen-presenting cells
HNC	Head and neck cancer
TCR	T cell receptor
PD-L1	Programmed cell Death 1 ligand 1
BTICs	Brain tumor-initiating cells
TSHR	Thyroid-stimulating hormone receptor
TSH	Thyroid-stimulating hormone
CRC	Colorectal cancer
Gal-1	Galectin-1
CCT2	Chaperonin-containing TCP1 subunit 2
GC	Gastric cancer
NPC	Nasopharyngeal carcinoma
TAM	Tumor-associated macrophages
LLC	Lewis lung carcinoma
TRIM59	Tripartite motif-containing 59
miR-21	microRNA-21
EMT	Epithelial–mesenchymal transition
OS	Osteosarcoma
TAFs	Tumor-associated fibroblasts
Ig	Immunoglobulin
TIM	T cell Ig and mucin domain
Breg	Regulatory B cells
TLR	Toll-like receptor
MAPK	Mitogen-activated protein kinase
IFN- γ	Interferon gamma
TANs	Tumor-associated neutrophils
NETs	Neutrophil extracellular traps
TF	Tissue factor
OC	Ovarian cancer
BCL2L13	BCL 2-like protein 13
CCA	Cholangiocarcinoma
CircRNAs	Circular RNAs
siRNA	Small interference RNA
NPC	Neural progenitor cells
BiP	Binding immunoglobulin protein
EC	Esophageal cancer
CAF	Cancer-associated fibroblasts
PDACs	Pancreatic adenocarcinomas
HS	Heparan sulfate
EGF	Epidermal growth factor
OSCC	Oral squamous cell carcinoma
V-type	Vacuolar-type
NSCLC	Non-small cell lung cancer
CKAP4	Cytoskeleton-associated protein 4
CLDN7	Claudin-7
